# Late Lyme Borreliosis Presenting with Chronic Painful Sensory Axonal Polyneuropathy Preceding Clinically Apparent Acrodermatitis Chronica Atrophicans: A Case Report

**DOI:** 10.3390/jcm15208030

**Published:** 2026-10-16

**Authors:** Fatima Elshafie Mahmoud Hassan, Hazem A. Juratli, Adrian Schmid

**Affiliations:** 1 Department of Acute Geriatric Medicine, Kantonsspital Winterthur, 8401 Winterthur, Switzerland; 2 Histological Diagnostics, Kempf and Pfaltz, Unilabs, 8050 Zurich, Switzerland; hazem.juratli@kempf-pfaltz.ch; 3 Infectious Diseases and Hospital Epidemiology, Department of General Internal Medicine, Kantonsspital Winterthur, 8401 Winterthur, Switzerland; adrian.schmid@ksw.ch

**Keywords:** Lyme borreliosis, acrodermatitis chronica atrophicans, Lyme neuroborreliosis, sensory axonal polyneuropathy, case report

## Abstract

**Background:** Lyme borreliosis is a multisystem tick-borne infection with heterogeneous clinical manifestations involving the skin, nervous system, joints, and other organs. Acrodermatitis chronica atrophicans (ACA) is the characteristic late cutaneous manifestation of European Lyme borreliosis and may be associated with peripheral neurological involvement. An atypical temporal sequence of neurological and cutaneous manifestations may delay diagnosis. **Case Presentation:** We report the case of a 78-year-old woman with more than one year of painful sensory symptoms affecting both feet and distal lower legs. Electroneurography demonstrated a sensory axonal polyneuropathy, and diagnostic investigations did not identify common metabolic, nutritional, autoimmune, infectious, and paraproteinaemic causes. Approximately 15 months later, she developed a progressive painful erythematous and oedematous lesion of the right lower leg. Histopathological findings were nonspecific and initially favoured interstitial granuloma annulare; however, PCR detected *Borrelia* DNA in the skin biopsy. Together with positive Borrelia serology and the clinical findings, this confirmed cutaneous Lyme borreliosis, with ACA considered the clinically favoured manifestation, although erythema migrans could not be definitively excluded. Cerebrospinal fluid analysis demonstrated lymphocytic pleocytosis and blood–CSF barrier dysfunction without intrathecal *Borrelia*-specific antibody synthesis. The overall findings supported a possible association between the sensory axonal polyneuropathy and Lyme borreliosis, although a coincidental unrelated polyneuropathy could not be excluded. Treatment with doxycycline resulted in near-complete resolution of the skin lesions and partial improvement of neuropathic pain. **Conclusions:** This case highlights the diagnostic challenges of late Lyme borreliosis presenting with chronic painful sensory axonal polyneuropathy preceding a cutaneous manifestation clinically considered most consistent with ACA by approximately 15 months. Although a direct causal relationship between the neuropathy and *Borrelia* infection cannot be definitively established, the overall clinicopathological findings support a possible association between the sensory axonal polyneuropathy and late Lyme borreliosis. Clinicians should consider late Lyme borreliosis in patients with otherwise unexplained painful sensory neuropathy when compatible cutaneous manifestations or other clinical features increase the pretest probability of Borrelia infection.

## 1. Introduction

Lyme borreliosis is the most common tick-borne infectious disease in Europe and North America and is caused by spirochetes of the *Borrelia burgdorferi* sensu lato complex, transmitted by *Ixodes* ticks. The clinical spectrum is broad and evolves from early localized infection, typically presenting with erythema migrans, to disseminated and late manifestations affecting the skin, nervous system, joints, and less frequently the heart. In Europe, several *Borrelia* species are pathogenic, with *Borrelia afzelii* predominantly associated with cutaneous manifestations such as acrodermatitis chronica atrophicans (ACA), whereas *Borrelia garinii* more commonly causes neuroborreliosis [1,2,3].

The epidemiology and clinical spectrum of Lyme borreliosis differ between Europe and North America. In North America, infection is predominantly caused by *B. burgdorferi* sensu stricto, whereas European Lyme borreliosis involves several pathogenic genospecies, particularly *B. afzelii* and *B. garinii.* Consequently, ACA and Bannwarth syndrome are encountered predominantly in Europe, whereas Lyme arthritis is a prominent manifestation in North America. These differences also influence the interpretation of serological assays and support the use of European diagnostic guidelines in the present case [1,2,3,4]. Climate change may further influence the epidemiology of Lyme borreliosis by expanding the geographical and altitudinal range of Ixodes ticks and prolonging periods of seasonal tick activity, potentially increasing opportunities for human exposure [5].

ACA is the characteristic late cutaneous manifestation of Lyme borreliosis in Europe and typically develops months to years after infection. Clinically, it begins with bluish-red discoloration and oedema of the distal extremities before progressing to cutaneous atrophy, fibrosis, and tissue thinning [1,6]. Histopathological findings are generally nonspecific. Serological testing remains the cornerstone of diagnosis, while detection of Borrelia DNA by PCR or isolation by culture from skin biopsy specimens can provide additional diagnostic support, particularly in atypical presentations. Current European guidelines recommend serological testing in all patients with suspected ACA and support oral antibiotic treatment for 21–28 days [3,6].

Neurological involvement occurs in approximately 3–15% of Lyme borreliosis infections and most commonly presents as polyradiculitis, meningitis, or cranial neuropathies, while myelitis, including transverse myelitis, and encephalomyelitis are rare manifestations [6,7]. Peripheral neuropathy is considerably less common and, in Europe, has been reported predominantly in association with ACA, typically presenting as an axonal polyneuropathy with predominantly sensory symptoms [8,9,10,11].

The diagnosis of Lyme neuroborreliosis is based on the integration of compatible neurological manifestations with laboratory findings. Current European and international guidelines recommend paired CSF and serum analysis in patients with suspected neuroborreliosis, including assessment of CSF inflammatory changes and *Borrelia*-specific intrathecal antibody production. Typical inflammatory CSF findings include lymphocytic pleocytosis, blood–CSF barrier dysfunction, and intrathecal immunoglobulin synthesis, although ACA-associated polyneuropathy is recognised as a possible exception to this typical CSF pattern [4,8]. Because *Borrelia*-specific antibodies may persist for years after infection or successful treatment, serological findings should be interpreted within the clinical context [1].

Although peripheral neuropathy associated with ACA has been recognised for several decades, reports describing PCR-confirmed ACA presenting with chronic sensory axonal polyneuropathy accompanied by inflammatory cerebrospinal fluid abnormalities remain uncommon. The simultaneous presence of these dermatological, neurological and cerebrospinal fluid findings poses a diagnostic challenge and may delay recognition of late Lyme borreliosis [4,8,12,13].

## 2. Case Presentation

### 2.1. Initial Neurological Presentation

A 78-year-old woman presented in March 2025 with progressive bilateral burning pain involving the feet and distal lower legs that had evolved over approximately three months, characterised by painful dysesthesias with nocturnal exacerbation. She denied motor weakness, gait disturbance, or sphincter dysfunction.

Her medical history included erosive osteoarthritis of the interphalangeal joints, osteoporosis treated with zoledronate, arterial hypertension, and previous total thyroidectomy for multinodular goitre. Regular medications included levothyroxine, acemetacin, vitamin D and calcium supplementation. She was a non-smoker and reported no alcohol use. There was no known family history of polyneuropathy or hereditary neurological disease.

Neurological examination demonstrated preserved muscle strength without focal neurological deficits. Sensory examination demonstrated distal burning dysesthesias with reduced vibration sensation at both ankles. Deep tendon reflexes were preserved except for an absent left Achilles tendon reflex. No cutaneous manifestations suggestive of Lyme borreliosis were reported at the initial neurological assessment; however, a dedicated dermatological examination of the lower extremities was not documented.

Nerve conduction studies performed in March 2025 revealed a predominantly sensory axonal polyneuropathy, with reduced sensory nerve action potential amplitudes and preserved conduction velocities, while motor nerve conduction studies were normal. Follow-up nerve conduction studies in April 2025 demonstrated persistent reduction in sensory nerve action potential amplitudes in the lower extremities with preserved motor nerve conduction. Repeat electrophysiological assessment in November 2025 showed a stable axonal polyneuropathy without evidence of significant progression (Table 1).

Laboratory evaluation for secondary causes of polyneuropathy included HbA1c, thyroid-stimulating hormone, vitamin B12 with holotranscobalamin, folate, renal function, serum protein electrophoresis with immunofixation and serum free light chains, serological testing for HIV, hepatitis B, hepatitis C, and syphilis, as well as antinuclear and antineutrophil cytoplasmic antibodies. No alternative cause of the polyneuropathy was identified. HbA1c was initially within the reference range and measured 5.8% on subsequent follow-up. The patient reported no alcohol use. Borrelia serology was not performed during the initial diagnostic work-up.

**Figure 1:**
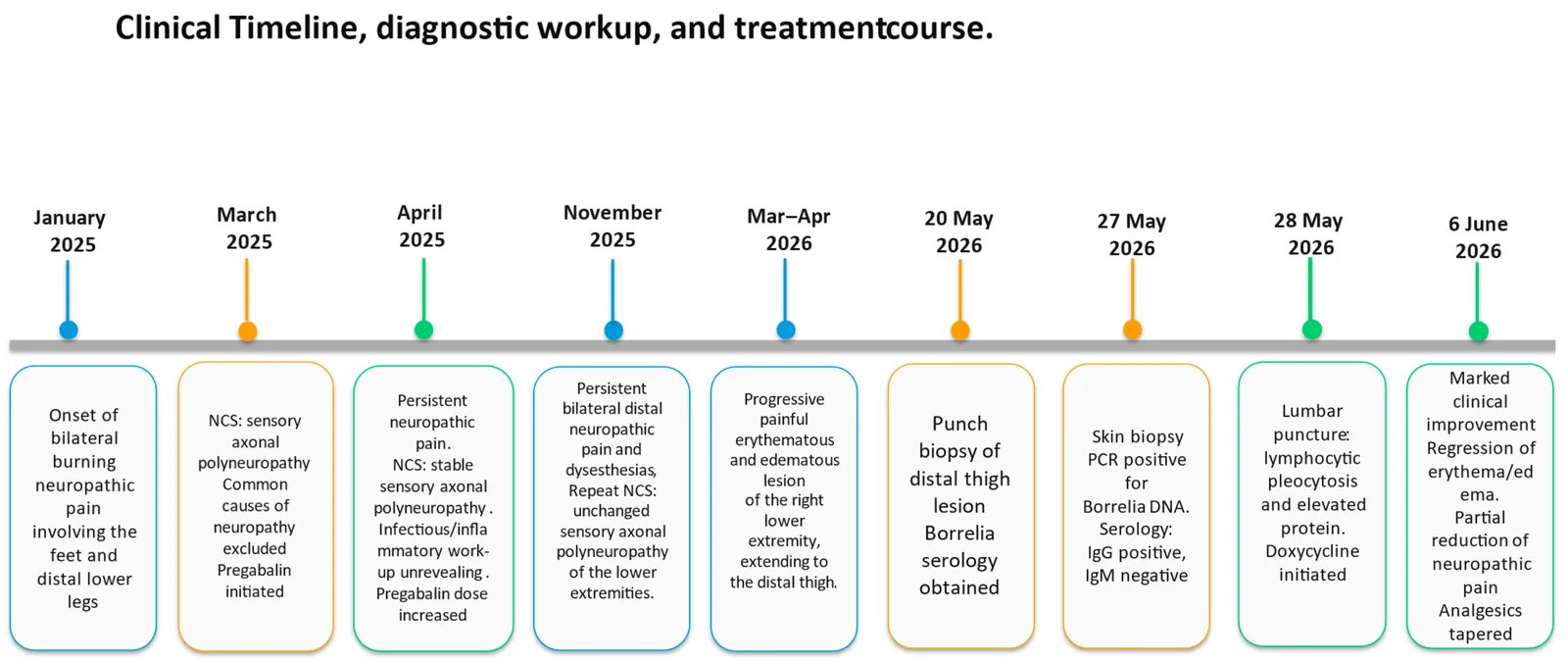
Clinical timeline, diagnostic workup, and treatment course. Abbreviations: NCS, nerve conduction studies; PCR, polymerase chain reaction.

Symptomatic treatment with pregabalin was initiated at 25 mg twice daily and subsequently titrated according to efficacy and tolerability to 50 mg during the day and 100 mg at night. Treatment resulted in minimal pain relief; however, neuropathic symptoms persisted. The clinical timeline, diagnostic workup, and treatment course are summarized in Figure 1.

### 2.2. Development of Cutaneous Manifestation

Approximately 15 months after the onset of neuropathic symptoms, the patient developed progressive erythema, swelling, and severe burning pain affecting the distal right lower extremity, with proximal extension to the distal thigh over several days. The patient recalled an erythema migrans following a tick bite approximately 20 years earlier, which had been treated with antibiotics. She did not recall any subsequent tick bite but reported regular gardening activities in a Lyme-endemic region.

**Figure 2:**
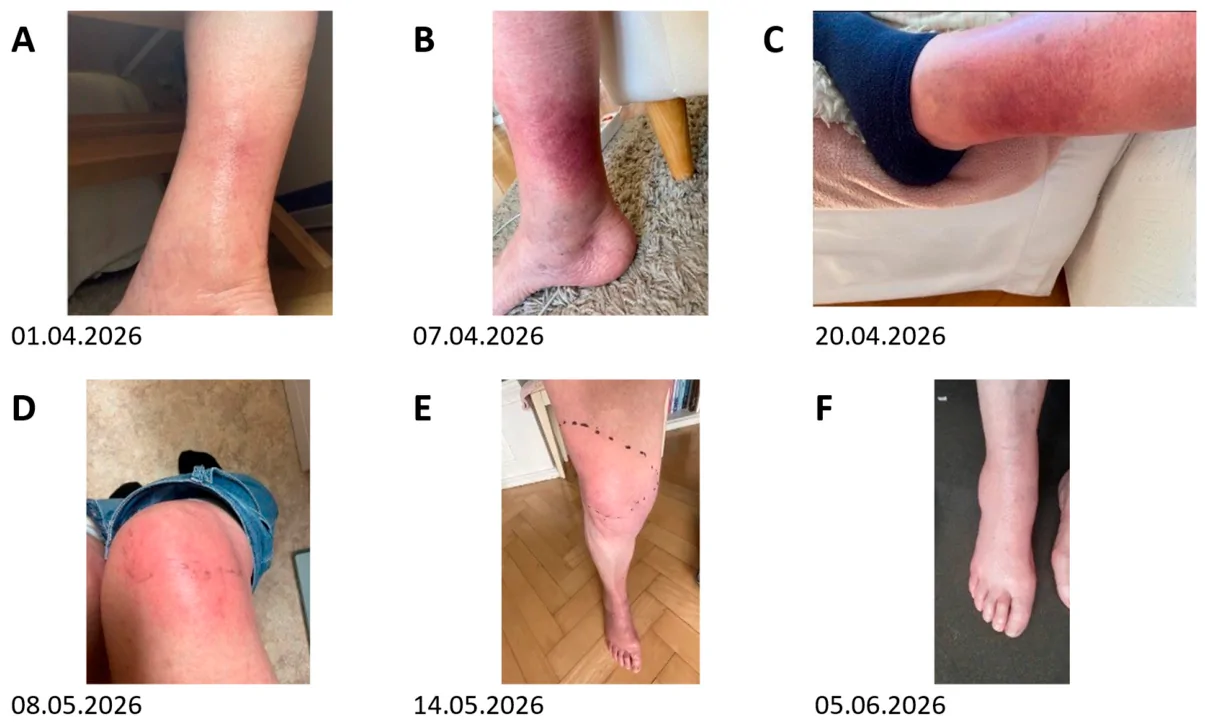
Evolution of cutaneous manifestations of the right lower extremity. (**A**–**E**) Progressive erythematous and oedematous cutaneous changes from April to May 2026, extending from the distal lower leg to the thigh. (**F**) Marked regression of the cutaneous inflammatory changes following antimicrobial treatment in June 2026.

Repeated duplex ultrasound examinations excluded deep vein thrombosis and thrombophlebitis. Angiological assessment did not reveal peripheral arterial disease or another vascular cause. Computed tomography of the right lower extremity demonstrated diffuse cutaneous thickening and subcutaneous oedema without evidence of abscess formation, osteomyelitis, or muscular involvement. Because severe pain and swelling were initially the predominant symptoms, the patient underwent sequential evaluations at different healthcare facilities to exclude vascular, infectious, and musculoskeletal causes. With progression of the skin changes despite these evaluations, dermatological assessment was subsequently obtained. The evolution of cutaneous manifestations and their subsequent regression following antimicrobial treatment are illustrated in Figure 2.

### 2.3. Diagnostic Evaluation of the Cutaneous Manifestation

During the following weeks, the progressive cutaneous changes prompted repeated medical evaluations before presentation to our hospital for further diagnostic assessment. Dermatological examination revealed a partially annular erythematous and oedematous lesion. Clinically, the differential diagnosis included erythema migrans and erysipelas. On 20 May 2026, a punch biopsy of the right thigh was obtained and Borrelia serology was performed. 

**Figure 3:**
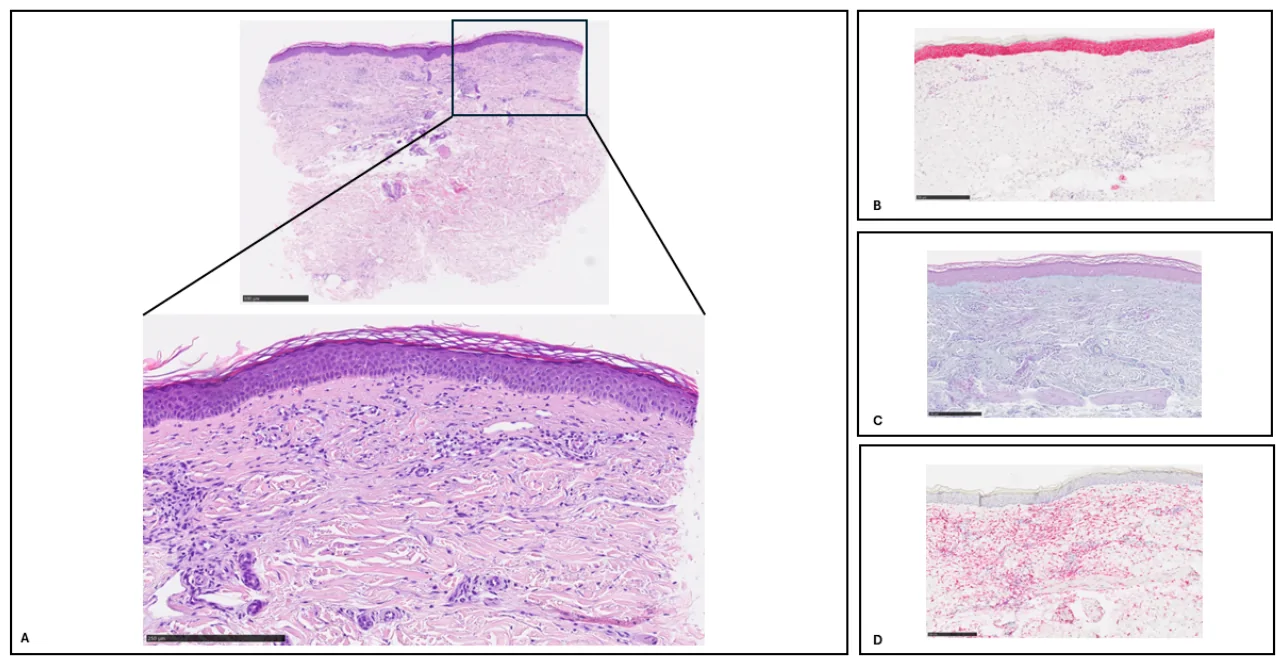
Histopathological findings of the skin biopsy. (**A**) Skin biopsy showing a basket-weave stratum corneum, mild epidermal flattening with effacement of the rete ridges, and a sparse diffuse interstitial dermal infiltrate predominantly composed of histiocytic cells (H&E, ×1.25 and ×4). (**B**) CD138 immunohistochemistry showing only isolated dermal plasma cells, accounting for <1% of the inflammatory infiltrate (×4). (**C**) Hale stain demonstrating increased interstitial mucin deposition (×4). (**D**) CD68 immunohistochemistry demonstrating strong positivity of the dermal histiocytic cells (×4).

Histopathological examination showed a sparse interstitial lymphohistiocytic infiltrate with predominantly CD68-positive histiocytes, isolated plasma cells on CD138 immunohistochemistry, and increased interstitial mucin deposition. The histopathological findings are illustrated in Figure 3. Overall, the findings were nonspecific and initially favoured interstitial granuloma annulare. Neither ACA nor erythema migrans could be established histologically, and further molecular testing for Borrelia was therefore recommended. Borrelia serology showed markedly elevated IgG antibodies (3354 AU/mL; reference < 10 AU/mL) with a positive IgG immunoblot, showing reactivity against VlsE BA, VlsE BB, VlsE BG, p41, p39, OspC, and p58; p83/100 was non-reactive. IgM antibodies were negative (2.8 AU/mL; reference < 18 AU/mL), as was the IgM immunoblot. In view of the positive serology, nested PCR of the skin biopsy was performed and detected *Borrelia* DNA, supporting the diagnosis of cutaneous Lyme borreliosis. Species identification was not performed. Based on the clinical presentation and overall disease course, the cutaneous manifestation was considered most consistent with acrodermatitis chronica atrophicans (ACA). However, given the nonspecific histopathological findings and absence of established cutaneous atrophy, erythema migrans could not be definitively excluded.

### 2.4. Evaluation for Neurological Involvement

Given the history of persistent painful sensory neuropathy and newly confirmed Lyme borreliosis, cerebrospinal fluid (CSF) analysis was performed to evaluate possible neuroborreliosis.

CSF examination demonstrated lymphocytic pleocytosis (51 leukocytes/µL; reference <5 leukocytes/µL), elevated protein concentration (869 mg/L) and blood–CSF barrier dysfunction with an albumin quotient of 14.9 on Reiber analysis. CSF glucose (3.3 mmol/L) and lactate (1.8 mmol/L) were within normal limits. Borrelia-specific antibodies were detectable in CSF; however, the Borrelia-specific antibody indices were not elevated (IgG antibody index 1.1; IgM antibody index 0.4; laboratory cutoff > 1.5). In the context of blood-CSF barrier dysfunction, this was interpreted as passive diffusion of Borrelia-specific antibodies into the CSF rather than intrathecal Borrelia-specific antibody synthesis.

Contrast-enhanced MRI of the brain showed no evidence of active central nervous system involvement, including no parenchymal lesions, meningeal enhancement, or cranial nerve abnormalities.

Based on the combination of chronic painful sensory axonal polyneuropathy, inflammatory CSF findings, and microbiologically confirmed cutaneous Lyme borreliosis with ACA as the clinically favoured manifestation, the overall findings supported possible Lyme-associated peripheral neurological involvement. However, a coincidental unrelated polyneuropathy could not be completely excluded.

### 2.5. Treatment and Outcome

The patient completed a 28-day course of doxycycline 100 mg orally twice daily, which was well tolerated without reported treatment-related adverse events. Supportive analgesic treatment was continued and gradually reduced.

Within two weeks after initiation of antimicrobial therapy, the patient showed clinical improvement, with partial improvement of neuropathic pain and regression of erythema and oedema of the right lower extremity. At follow-up, the cutaneous inflammatory changes had largely resolved, while neuropathic pain remained partially improved.

At neurological follow-up approximately three months after completion of antibiotic therapy, the swelling and erythema of the right lower extremity had markedly regressed. Although neuropathic pain persisted, with allodynia and nocturnal exacerbations, its overall intensity had decreased compared with the acute phase, accompanied by substantial reduction in opioid requirements. Pregabalin was continued for neuropathic pain. Neurological examination showed preserved strength, normal gait, and bimalleolar vibration perception of 8/8. No repeat lumbar puncture was considered clinically indicated. Despite the reduction in overall pain intensity, persistent neuropathic symptoms continued to substantially affect her quality of life. No validated pain score was recorded, and repeat nerve conduction studies were not performed during follow-up. 

### 2.6. Patient Perspective

The patient expressed relief that, after a prolonged period of symptoms and diagnostic uncertainty, a diagnosis had finally been established and appropriate antimicrobial treatment administered. Although pain intensity decreased following treatment, she continued to experience neuropathic pain, particularly at night, with persistent impairment of her quality of life. She reported trying to accept and adapt to the persistent symptoms.

## 3. Discussion

This case illustrates an unusual presentation of late Lyme borreliosis in which chronic painful sensory axonal polyneuropathy preceded the clinically apparent cutaneous manifestation considered most consistent with acrodermatitis chronica atrophicans (ACA) by approximately 15 months. Although both peripheral neuropathy and ACA are recognised manifestations of late Lyme borreliosis, this atypical sequence delayed recognition of underlying infection because the patient initially presented without characteristic cutaneous manifestations of Lyme borreliosis [6,9]. The diagnosis of cutaneous Lyme borreliosis was supported by positive serology and detection of Borrelia DNA in the skin biopsy. ACA was considered the most likely cutaneous manifestation based on the overall clinical presentation and disease course, although erythema migrans could not be excluded with certainty. 

Peripheral neuropathy is an uncommon neurological manifestation of Lyme borreliosis and, in Europe, has been reported predominantly in association with ACA, typically as an axonal polyneuropathy with predominantly sensory symptoms. The underlying mechanisms are thought to involve both direct tissue infection and the host inflammatory response, although the pathogenesis of chronic peripheral nerve injury remains incompletely understood [8,10,14,15].

An important clinical lesson from this case is that Lyme borreliosis may not be considered in patients presenting with otherwise unexplained painful sensory polyneuropathy before the appearance of a compatible cutaneous manifestation. In our patient, the initial diagnostic work-up did not identify an alternative cause of the sensory axonal polyneuropathy, and no clinical features initially suggested Lyme borreliosis. Consequently, Borrelia serology was not performed until the cutaneous manifestation developed approximately 15 months later. As ACA may arise months to years after the initial infection, clinicians should reconsider late Lyme borreliosis when otherwise unexplained painful sensory neuropathy is subsequently accompanied by compatible dermatological findings, particularly in endemic regions [2,6,16].

The differential diagnosis of peripheral neuropathy associated with lower-limb skin discoloration is broad. Diabetes and common nutritional causes were not identified in the diagnostic work-up. Autoimmune and vasculitic causes were considered less likely given negative ANA/CTD screening and PR3- and MPO-ANCA. Serum protein electrophoresis and immunofixation were unremarkable, with a normal serum free light-chain ratio, providing no evidence of a monoclonal gammopathy and making plasma-cell disorders such as POEMS syndrome or AL amyloidosis less likely. Cryoglobulinaemia was not specifically tested. Angiological assessment showed no deep-vein thrombosis, thrombophlebitis, lymphoedema, or clinically relevant venous disease that could explain the progressive skin changes. There were no clinical features suggestive of sarcoidosis or leprosy. Nevertheless, chronic idiopathic axonal polyneuropathy remains an alternative explanation of the neurological findings.

The histopathological findings in this case were nonspecific and initially favoured interstitial granuloma annulare. This diagnostic challenge is relevant because evolving ACA may show an interstitial inflammatory pattern that can overlap histologically with other inflammatory dermatoses. However, erythema migrans could not be excluded on histopathological grounds, and in view of the clinical differential diagnosis, molecular testing was recommended. PCR subsequently detected *Borrelia* DNA in the skin biopsy, providing direct microbiological evidence of cutaneous *Borrelia* infection. This case therefore illustrates the importance of clinicopathological correlation and adjunctive molecular testing when the histopathological findings of suspected cutaneous Lyme borreliosis are inconclusive [17,18].

The distinction between ACA and erythema migrans warrants particular consideration in this case. The relatively rapid evolution of the cutaneous changes, absence of established cutaneous atrophy, and paucity of plasma cells on histopathological examination are not classical features of fully developed ACA and may also be compatible with erythema migrans. However, ACA can initially present with an oedematous-infiltrative inflammatory phase before development of overt cutaneous atrophy [6]. In the present case, the treating multidisciplinary team considered ACA the more likely clinical manifestation based on the overall clinical presentation and disease course. Nevertheless, the available clinicopathological findings do not allow erythema migrans to be excluded with certainty.

Serological testing remains the cornerstone of laboratory diagnosis in Lyme borreliosis. Current guidelines recommend validated two-tier serological testing algorithms, while serological findings must always be interpreted within the clinical context because Borrelia-specific antibodies may persist for many years after infection and therefore cannot distinguish active infection from previous exposure. In longstanding manifestations such as ACA, positive IgG serology provides supportive evidence when interpreted in conjunction with compatible clinical findings [1,3,19]. In our patient, positive serology together with detection of Borrelia DNA in the skin biopsy established cutaneous Lyme borreliosis; however, these findings did not independently distinguish ACA from erythema migrans.

The principal diagnostic dilemma in this case was whether the chronic sensory axonal polyneuropathy represented neurological involvement of Lyme borreliosis. Diagnostic classification depends on the clinical context and guideline framework. According to the EFNS criteria, Lyme neuroborreliosis is classified as definite when all three criteria are fulfilled—neurological symptoms suggestive of Lyme neuroborreliosis, CSF pleocytosis, and intrathecal production of Borrelia-specific antibodies—and as possible when two of three criteria are fulfilled [4]. In our patient, neurological symptoms and CSF pleocytosis were present, but intrathecal Borrelia-specific antibody synthesis was not demonstrated; the findings therefore fulfilled EFNS criteria for possible, but not definite, Lyme neuroborreliosis. More recent DGN criteria similarly distinguish possible, probable, and confirmed Lyme neuroborreliosis, with confirmed disease requiring intrathecal Borrelia-specific antibody synthesis or direct pathogen detection in CSF [8]. The DGN guideline additionally considers polyneuropathy associated with ACA as a distinct clinical context. In the present case, the inflammatory CSF profile in this patient is noteworthy because ACA-associated polyneuropathy may occur without CSF inflammation. Accordingly, the pleocytosis and blood-CSF barrier dysfunction should not be regarded as specific evidence linking the polyneuropathy to Lyme borreliosis, and other inflammatory neurological processes remain part of the differential diagnosis.

Previous studies have described heterogeneous patterns of peripheral neuropathy associated with acrodermatitis chronica atrophicans (ACA), including symmetric distal sensory polyneuropathy as well as neuropathy localized to affected extremities (Table 2). The symmetric, length-dependent neuropathy observed in our patient therefore falls within the reported spectrum, although its distribution differed from the localized or asymmetric patterns described in some patients with ACA-associated neuropathy.

These studies demonstrate that ACA-associated peripheral neuropathy is heterogeneous and that neurological improvement following antimicrobial treatment may be incomplete. 

An important question remains whether the chronic sensory axonal polyneuropathy represented a neurological manifestation of Lyme borreliosis or an unrelated coincidental neuropathy. Because *Borrelia* investigations were not performed when the neurological symptoms first developed, a definite causal relationship cannot be established retrospectively. Conversely, several observations favour an association with Lyme borreliosis, including the predominantly sensory axonal phenotype, inflammatory CSF findings, microbiologically supported cutaneous Lyme borreliosis with ACA as the clinically favoured manifestation, and partial improvement of neuropathic symptoms following antimicrobial therapy. Although none of these findings independently establish causality, their combination supports a possible association between the polyneuropathy and Lyme borreliosis, while an unrelated coincidental neuropathy cannot be completely excluded.

The 28-day course of oral doxycycline was selected primarily for treatment of the clinically suspected ACA, in accordance with recommended treatment regimens for this manifestation. Oral doxycycline is also an established treatment option for European Lyme neuroborreliosis without parenchymal central nervous system involvement [6,8].

Following treatment with doxycycline, the patient experienced regression of the cutaneous lesions together with partial improvement of neuropathic pain, allowing reduction in analgesic therapy. Clinical improvement after antibiotic treatment has been reported in patients with neurological manifestations of Lyme borreliosis, although recovery may be incomplete, particularly when symptoms have been present for a prolonged period before treatment [1,6]. In the present case, the partial improvement following antimicrobial therapy was compatible with, but does not establish, a causal relationship between the polyneuropathy and Lyme borreliosis.

This case has several limitations. First, Borrelia serology and CSF analysis were performed only after the appearance of the cutaneous manifestation, precluding assessment of Lyme-associated neurological involvement at symptom onset. Second, small-fiber neuropathy-specific testing, hereditary neuropathy testing, and paraneoplastic antibody screening, oral glucose testing, vitamin B6 and copper measurements, and specific anti-SSA/SSB antibody testing were not performed. Third, despite lymphocytic pleocytosis and blood-CSF barrier dysfunction, intrathecal Borrelia-specific antibody synthesis was not demonstrated, preventing classification as confirmed Lyme neuroborreliosis according to current diagnostic criteria. In addition, CSF PCR for Borrelia was not performed. Finally, although the clinically favoured diagnosis of ACA, inflammatory CSF findings, absence of an identified alternative cause in the diagnostic work-up, and partial improvement following antimicrobial therapy support a possible association between the polyneuropathy and Lyme borreliosis, a coincidental sensory axonal polyneuropathy cannot be entirely excluded.

## 4. Conclusions

This case highlights the diagnostic challenges of late Lyme borreliosis presenting with an atypical sequence of manifestations. Chronic painful sensory axonal polyneuropathy preceded the clinically apparent cutaneous manifestation considered most consistent with acrodermatitis chronica atrophicans (ACA) by approximately 15 months, illustrating that neurological symptoms may occur before characteristic cutaneous findings become apparent. Although a direct causal relationship between the sensory axonal polyneuropathy and *Borrelia* infection cannot be definitively established, the combination of microbiologically supported cutaneous Lyme borreliosis with ACA as the clinically favoured manifestation, inflammatory cerebrospinal fluid findings, absence of an identified alternative cause of polyneuropathy, and partial improvement following antimicrobial therapy supports a possible association between the sensory axonal polyneuropathy and late Lyme borreliosis. In patients with otherwise unexplained painful sensory neuropathy, Lyme borreliosis may be considered in the differential diagnosis when supported by compatible epidemiological exposure, clinical features, or subsequent characteristic manifestations. Recognition of the diverse clinical presentations of late Lyme borreliosis and careful integration of dermatological, neurological, histopathological, microbiological, serological, and cerebrospinal fluid findings are essential for timely diagnosis and appropriate treatment.

## Figures and Tables

**Table 1:** Serial nerve conduction study findings during follow-up.

Date	Nerve	Latency (ms)	Amplitude	Conduction Velocity m/s	Interpretation
**13 March 2025**	Sural sensory, left	2.70	0.84 µV	48.6	Reduced SNAP amplitude
	Superficial peroneal sensory, left	2.98	0.53 µV	48.3	Reduced SNAP amplitude
	Ulnar sensory, left	3.39	0.79 µV	54.0	Reduced SNAP amplitude
	Peroneal motor, left	4.48	1.47 mV	46.8	Normal motor study
	Tibial motor, left	4.75	3.4 mV	49.1	Normal motor study
	Ulnar motor, left	2.78	4.9 mV	55.6	Normal motor study
**29 April 2025**	Sural sensory, left	1.86	1.80 µV	41.9	Reduced SNAP amplitude
	Sural sensory, right	3.57	1.52 µV	43.2	Reduced SNAP amplitude
	Ulnar sensory, left	3.33	2.0 µV	53.1	Low SNAP amplitude
	Peroneal motor, left	4.00	2.0 mV	47.0	Normal motor study
	Tibial motor, left	3.92	4.7 mV	46.5	Normal motor study
	Ulnar motor, left	2.75	6.1 mV	62.1	Normal motor study
**13 November 2025**	Sural sensory, left	3.12	0.83 µV	46.2	Reduced SNAP amplitude
	Superficial peroneal sensory, left	NR	NR	NR	No measurable response documented
	Ulnar sensory, left	3.35	9.5 µV	60.5	Preserved sensory response
	Tibial motor, left	4.21	5.1 mV	47.6	Normal motor study
	Ulnar motor, left	2.43	9.6 mV	65.6	Normal motor study

Abbreviations: CMAP, compound muscle action potential; SNAP, sensory nerve action potential; NR, no measurable response documented. Note: For motor studies, latency and amplitude refer to distal stimulation; conduction velocity represents the principal measured motor segment. Laboratory reference limits were provided in the original reports for motor nerve conduction parameters but not for sensory SNAP amplitudes or sensory conduction velocities. Sensory studies are therefore classified according to the interpretation provided in the original electrophysiological reports.

**Table 2:** Selected studies of peripheral neuropathy associated with acrodermatitis chronica atrophicans (ACA).

No.	Study	Study Population	Neuropathy Phenotype/Findings	CSF Findings	Treatment and Outcome
1	Kristoferitsch et al., 1988 [20]	Patients with ACA-associated neuropathy	Clinical, electrophysiological, and morphological characterization of peripheral neuropathy associated with ACA	Not reported in the available publication data	Not reported in the available publication data
2	Kindstrand et al., 1997 [21]	63 consecutive untreated patients with ACA	Clinical and/or neurophysiological signs of peripheral neuropathy in 64%; symmetric distal sensory polyneuropathy was the most common phenotype; pain and/or paraesthesia were common	Not reported in the available publication data	Treatment outcome not assessed
3	Kindstrand et al., 2000 [22]	17 patients with ACA-associated polyneuropathy	Prospective clinical and neurophysiological characterization of polyneuropathy associated with ACA	Not reported in the available publication data	Treatment outcome not the primary focus
4	Kindstrand et al., 2002 [23]	47 patients with ACA and objective neurological and/or neurophysiological abnormalities	Peripheral nerve abnormalities associated with ACA	Not reported in the available publication data	ACA lesions and symptoms of irritative nerve involvement improved after antibiotic treatment, whereas objective neurological and neurophysiological deficits remained unchanged
5	Ogrinc et al., 2021 [24]	693 patients with ACA	Symptoms and/or signs suggestive of peripheral neuropathy in 20.8%; these were confined to sites affected by ACA	Not systematically reported	Treatment outcome of neuropathy not assessed in this cohort

## Data Availability

All relevant information supporting this case report is provided within the published article. Any additional inquiries may be addressed to the corresponding author.
